# Understanding Progressive Multifocal Leukoencephalopathy Risk in Multiple Sclerosis Patients Treated with Immunomodulatory Therapies: A Bird’s Eye View

**DOI:** 10.3389/fimmu.2018.00138

**Published:** 2018-02-02

**Authors:** Elizabeth A. Mills, Yang Mao-Draayer

**Affiliations:** ^1^Department of Neurology, University of Michigan Medical School, Ann Arbor, MI, United States; ^2^Graduate Program in Immunology, Program in Biomedical Sciences, University of Michigan Medical School, Ann Arbor, MI, United States

**Keywords:** John Cunningham virus, natalizumab, fingolimod, dimethyl fumarate, glia

## Abstract

The increased use of newer potent immunomodulatory therapies for multiple sclerosis (MS), including natalizumab, fingolimod, and dimethyl fumarate, has expanded the patient population at risk for developing progressive multifocal leukoencephalopathy (PML). These MS therapies shift the profile of lymphocytes within the central nervous system (CNS) leading to increased anti-inflammatory subsets and decreased immunosurveillance. Similar to MS, PML is a demyelinating disease of the CNS, but it is caused by the JC virus. The manifestation of PML requires the presence of an active, genetically rearranged form of the JC virus within CNS glial cells, coupled with the loss of appropriate JC virus-specific immune responses. The reliability of metrics used to predict risk for PML could be improved if all three components, i.e., viral genetic strain, localization, and host immune function, were taken into account. Advances in our understanding of the critical lymphocyte subpopulation changes induced by these MS therapies and ability to detect viral mutation and reactivation will facilitate efforts to develop these metrics.

## Introduction

Progressive multifocal leukoencephalopathy (PML) is a rare polyomavirus-associated disease involving progressive damage to brain white matter that often results in permanent disability or death. PML was first characterized in the 1950s in immunocompromised patients with lymphoproliferative disorders (1) and has come to be understood as an opportunistic infection associated with immunosuppression. The disease is caused by the infection, and subsequent loss of glial cells, especially myelin-producing oligodendrocytes, by a mutated form of the John Cunningham virus (JCV). Most people acquire JCV, usually in childhood, as it is found in 70–90% of the population. The initial infection is thought to occur in the tonsils or gastrointestinal tract, and then the virus remains latent, often in the kidneys or lymphoid organs, in an archetypal form that is incapable of productively infecting glial cells (2). Immunosuppression can lead to the reactivation of the latent virus and may promote viral mutation, thereby facilitating infection of glia by viral strains with mutated regulatory regions (3). Continued immunosuppression then prevents clearance of the virus from infected glia, resulting in demyelination and neurodegeneration.

The AIDS epidemic of the 1980s led to a dramatic increase in new cases of PML, which helped fuel research toward the mechanism, risk factors, diagnostic markers, and possible therapeutic interventions for this devastating disease (4). Unfortunately, while survival rates have increased, there has not been enough progress in the prevention of PML, and many immunocompromised individuals remain at risk. The increased availability of antiretroviral therapies for HIV patients has not decreased rates of HIV-associated PML as much as anticipated (5). Furthermore, the recent widespread use of next-generation immunomodulatory treatments for autoimmune diseases such as multiple sclerosis (MS) has expanded the patient population at an increased risk for the development of PML.

The immunomodulatory drugs natalizumab, fingolimod, and dimethyl fumarate (DMF) have greater efficacy in reducing relapses for many relapsing-remitting MS (RRMS) patients than older treatments, such as interferon-β and glatiramer acetate, but have also led some patients to develop PML (6). Natalizumab was the first of these therapies to be tied to PML in MS patients, and, of all MS therapies, it has the highest global incidence for PML (7). The inability to accurately predict risk can lead to difficult treatment decisions, particularly in patients with highly active MS, which need to switch therapies. The off-label use of the chimeric anti-CD20 monoclonal antibody, rituximab, has not been associated with PML in MS patients. Consequently, MS patients who develop PML while taking other therapies, particularly natalizumab, are often switched to rituximab to prevent worsening of either MS or PML (8). However, cases of PML have been linked to the use of rituximab in other autoimmune conditions such as rheumatoid arthritis (9). The reasons for the discrepancy are not fully understood but may be related to the combinatorial use of rituximab with other immunosuppressive agents. The risk of PML for MS patients taking anti-CD20 therapy with a history of taking natalizumab remains unclear and merits further study. This is likely to become an increasingly important factor in future treatment decisions, as the humanized anti-CD20 antibody, ocrelizumab, has recently been approved by the FDA for RRMS and primary progressive MS.

The most commonly used clinical metric for identifying patients at risk for developing PML is absolute lymphocyte count. Unfortunately, this metric has not been a reliable predictor. Indeed due to its mechanism of action, fingolimod generally lowers lymphocyte counts to a greater extent than natalizumab or DMF (10–12), but has a lower incidence of PML (7). Efforts have been made to determine the factors associated with increased risk, but thus far there are no truly predictive measures available.

Three factors identified to help stratify the pool of natalizumab-treated MS patients at greatest risk for developing PML include the presence of JCV antibodies in patient’s serum, previous use of immunosuppressive drugs, and use of natalizumab exceeding 24 months (13). A patient with all three factors has a 2.3% risk of developing PML, while a patient with none of the risk factors has a 0.002% risk (14). JCV blood antibody index can further stratify this risk, with an antibody index above 1.5 associated with a higher risk (15). Determining whether a patient is seropositive for JCV prior to the start of treatment is recommended; however, the high rate of false negatives in these assays can skew risk assessments (16). In addition, patients seroconvert at higher frequencies during treatment (17, 18). Therefore, it will be necessary to monitor additional parameters throughout the course of treatment to more accurately determine risk.

**Figure 1:**
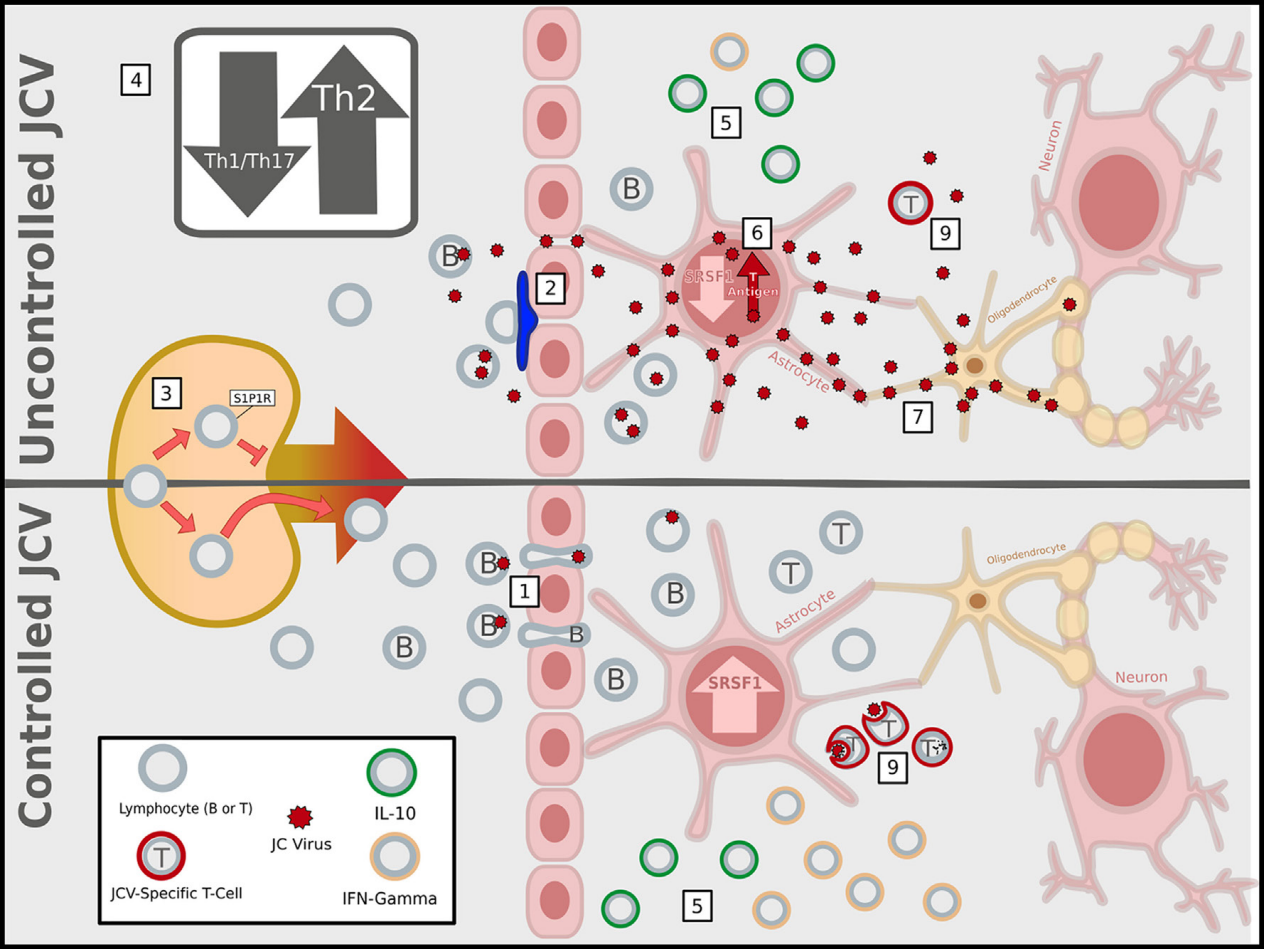
Immunosuppressive activities of multiple sclerosis (MS) therapies facilitate John Cunningham virus (JCV) infection and replication in the central nervous system (CNS). MS immunomodulatory therapies associated with progressive multifocal leukoencephalopathy have different mechanisms of action, but ultimately lead to an immunosuppressed state within the CNS that increases the likelihood of a productive infection of glial cells by JCV, represented as the uncontrolled JCV state. (1) In a healthy immune system (or the absence of immunomodulatory therapy), lymphocytes can enter the CNS *via* the blood–brain barrier, blood–meningeal barrier, or blood–cerebrospinal fluid barrier (latter two not shown), and JCV infected B-cells have been proposed as carriers of the virus into the CNS. In contrast, MS therapies block CNS entry of specific lymphocyte subsets: (2) natalizumab prevents CNS access of α4 integrin expressing lymphocytes primarily across the BBB by blocking α4β1/vascular cell adhesion molecule 1 (VCAM-1) adhesion interactions; (3) fingolimod traps within lymph nodes the lymphocytes that utilize sphingosine-1-phosphate 1 (S1P1) receptors for homing; (4) dimethyl fumarate interferes with the maturation of Th1 T-lymphocytes, tipping the balance in favor of anti-inflammatory Th2 cells. (5) Within the CNS, the net effect reduced the entry of conventional lymphocytes secreting pro-inflammatory IFN-γ and a relatively higher percentage of interleukin-10 (IL-10)-producing anti-inflammatory regulatory lymphocytes, compared to the healthy/untreated state. (6) The altered cytokine profile affects cross-talk between lymphocytes and CNS-resident astrocytes leading to transcriptional changes, such as the suppression of SRSF1, which can promote viral T-antigen expression, reactivation, and replication. (7) The JCV-infected astrocytes could then pass on the virus to oligodendrocytes and (8) fail to properly recruit the subset of lymphocytes necessary to clear the virus. (9) While most are blocked, the JCV specific T-cells that are present fail to adequately clear JCV (Image copyright: Caitlyn Fisher and Yang Mao-Draayer, reprints use with permission).

Since PML has occurred in the context of several MS disease-modifying therapies, a better understanding of how PML develops under each of these conditions could lead to the identification of universal predictive risk factors, as well as those factors that are unique. Recent work suggests that efforts toward the combined monitoring of specific lymphocyte subsets, JCV reactivation, and viral genomic rearrangements within the central nervous system (CNS) may offer better insight toward predicting risk (see Figure 1).

## Immune Cell Function

Lymphopenia is a PML risk factor, but simply monitoring absolute lymphocyte count does not accurately convey risk, because it does not take into account the diversity and complexity of the immune system. MS therapies have been designed to ameliorate the inflammatory overresponse to autoantigens (19). This inflammatory response toward myelin-associated proteins results in the demyelinating lesions that are hallmarks of the disease. Consequently, MS treatments do not affect all immune cell types equally, but rather attempt to restore the balance toward a more anti-inflammatory state (20). This can lead to alterations in specific critical immune cell subtypes without dramatic changes to the overall lymphocyte count. Since the immune system is composed of a multitude of cell types, which have unique roles in maintaining proper immune function, the loss of particular subsets of lymphocytes can disproportionately elevate PML risk. For instance, CD4^+^ T-cell lymphocytopenia has been associated with PML in the context of HIV (21); however, simply monitoring absolute changes in a single specific subset is also insufficient. Instead, changes in the relative distribution and function of specific immune cells are critical to both the therapeutic benefit toward MS and the potential risk of developing PML.

The shift toward a more anti-inflammatory environment is driven, in part, by changes in the balance between conventional and regulatory immune cells in number and/or function. Regulatory cells can dampen the response of conventional immune cells, thereby suppressing the immune system (22). In some cases, the balance may be shifted too far, resulting in chronic immunosuppression and increasing the risk for opportunistic infections. In MS patients, the function of regulatory cells is thought to be compromised, leading to an exaggerated inflammatory response (23–25). The cytokine interleukin-10 (IL-10) is a critical anti-inflammatory mediator, which is decreased in RRMS patients (26). Many effective RRMS therapies have been found to increase the number or response of regulatory cells (20) and levels of IL-10 (27). It is important to note, however, that there is no correlation between MS therapies, which affect IL-10 and the subset associated with PML. Consequently, changes in IL-10 levels alone are not prognostically useful in risk assessments for PML.

Many pathogens, particularly those which can remain latent within B-cells, exploit the immunosuppressive properties of IL-10 to promote their persistence (28). These mechanisms are especially prevalent among viruses that are associated with chronic infections and immune exhaustion. Some viruses, such as human cytomegalovirus and Epstein–Barr virus (EBV), encode for an IL-10 ortholog within their viral genome (29), whereas others, such as hepatitis B virus, increase the expression of cellular IL-10 (30). The net effect of these strategies is to alter the balance of the endogenous cytokine secretions of immune cells toward an anti-inflammatory state. In some strains of mice, polyomaviruses are associated with tumor formation, and susceptibility appears to be related to the aberrant production of IL-10 in response to the virus in affected strains (31). A similar aberrant IL-10 response to JCV antigens by JCV-specific T-cells has been found in some PML patients, which facilitates the maintenance rather than the clearance of the virus (32, 33). This inappropriate response is typically a feature of exhausted immune cells in a chronically immunosuppressed environment (34). Furthermore, elevated cerebrospinal fluid (CSF) levels of IL-10 have been detected in about half of early stage PML patients (33). Consequently, treatments that increase levels of IL-10 are therapeutically beneficial in the context of MS, but elevated IL-10, particularly in the CNS, may also prevent antigen-specific T-cells from appropriately responding to JCV.

Chronic infections involving the continued presence of antigen lead to immune exhaustion, resulting in the eventual inability of T-cells to mount an effective response to the persisting antigen (35). In some cases, the development of an exhausted T-cell response can represent a “compromise” between pathogen and host, which allows for maintenance of the virus without incurring widespread tissue damage (36). Under these circumstances, attempts to restore T-cell responses can inflict great harm to the host, but in the context of other pathogens, restoration of immune responses can be beneficial. Thus, it is crucial to understand the relationship between immune exhaustion and pathogenesis for a given virus when developing or administering potential immune boosting therapies. Achieving the correct balance is a particularly challenging endeavor in the context of autoimmune diseases, since immune suppression promotes exhaustion both toward autoantigens and foreign antigens.

Sustained surface expression of the inhibitory receptor programmed death-1 (PD-1) is associated with T-cell exhaustion, and blockade of PD-1 can help restore immune responsiveness (37). Relative to the total population of CD8^+^ T-cells, elevated levels of PD-1 have been found on the JCV-specific CD8^+^ T-cells of PML patients and are associated with the lack of functional response to JCV peptides (38). The results of an *in vitro* assay suggest that blocking PD-1 could help enhance JCV-specific CD8^+^ T-cell responses in a subset of PML patients, especially those at early stages (38). However, this particular experiment involved patients with HIV-associated PML, rather than immunomodulatory therapy-induced PML, and the use of anti-PD-1 therapy may exacerbate autoimmune diseases. Similar to IL-10, dysfunction of PD-1 is associated with MS, such that polymorphisms that decrease PD-1 function are linked to disease progression and severity (39, 40). Furthermore, these data suggest that targeting PD-1 may only be clinically useful in a specific subset of early PML patients and that monitoring PD-1 expression alone is unlikely to be a reliable predictive or diagnostic metric. As PD-1 is only one of several co-stimulatory receptors associated with immune exhaustion, a reliable assay will likely require the combined analysis of multiple markers.

Notably, in the context of murine polyomavirus, both susceptible and resistant mouse strains produce a similar antibody response, but differ in the strength of their antigen-specific cytotoxic T-lymphocyte (CTL) responses (41). In the process of co-opting B-cells as a host, EBV promotes B-cell proliferation and antibody production, with high antibody titers associated with chronic uncontrolled infection, rather than immunity (42). A similar situation may occur in JCV-infected B-cells, thereby explaining the seemingly paradoxical clinical finding that higher levels of anti-JCV antibodies are associated with greater risk in the context of PML (15), as there is a reason to believe that the infection of B-cells could be a significant risk factor for PML.

B-cells have been implicated as carriers of JCV from the periphery into the CNS, since they can be non-productively infected by JCV and serve as reservoirs of latent virus (43). The form of JCV associated with PML has a rearranged non-coding regulatory region (NCCR) and often contains binding sites for the transcription factor Spi-B, which promotes expression of early genes in the JCV genome (44). Similar to EBV, JCV may induce pathogenic mutations within its viral genome through activation of class-switch recombinases in B-cells, as the JCV NCCR contains putative recombination sites (45). Viral homology-based recombination can also occur in B-cells co-infected with EBV and JCV and provides another potential avenue for pathogenic rearrangement of JCV (46). Furthermore, because Spi-B is also important for proper B-cell maturation and function (47), therapies that affect the distribution or expression profile of B-cells could facilitate viral reactivation and spread. Indeed, natalizumab promotes B-cell differentiation-associated gene expression (48), including increased expression of Spi-B in CD19^+^ B-cells (49). It is likely that the subpopulation of B-cells infected by the virus is also of critical consequence, with some stages having a more detrimental effect on subsequent immune responses and viral transmission. The rearrangement of immunoglobulin genes through V(D)J recombination in pre-B-cells makes this population an attractive candidate to facilitate JCV NCCR rearrangement (50). Notably, circulating pre-B-cells also increase in response to natalizumab treatment (51, 52). Therefore, the use of diagnostic tests for JCV specifically within B-cells may help further stratify patients at greatest risk for PML.

As a disease of the CNS, there needs to be a correlation between peripheral and central changes for peripheral lymphocyte monitoring to have predicative and diagnostic merit for PML. Since each drug has a unique mechanism of action, the lymphocyte populations affected, and the cell subtype ratio changes that are most predictive of PML will also likely vary based on the treatment history of the patient. In addition, changes in the relative frequencies and function of lymphocyte subsets may vary overtime with a given therapy. Although accurate predictive metrics for PML are still lacking, recent advances in our understanding of how these next-generation MS therapies differentially affect lymphocyte populations may allow for the development of novel assays and monitoring guidelines with increased predictive power.

### Natalizumab

#### Mechanism

Natalizumab is a recombinant humanized monoclonal immunoglobulin G4 antibody targeting α-4 integrin (CD49d) that has been approved for the treatment of RRMS and Crohn’s disease (53). Natalizumab treatment leads to CD49d downregulation in CD49d-expressing cells (54). Very late antigen-4 (VLA-4) is a heterodimer composed of CD49d and β-1 integrin (α4β1 integrin) found on several immune cell populations, which interacts with vascular cell adhesion molecule 1 (VCAM-1) on endothelial cells and facilitates migration across endothelial barriers, including the blood–brain barrier (BBB) (55). VLA-4/VCAM-1 is one of the several adhesive molecule interactions that mediate CNS entry, and thus loss of this interaction will diminish, but not abolish, lymphocyte migration into the CNS. The other most prominent interaction is between lymphocyte function-associated antigen 1 (LFA-1) and endothelial intercellular adhesion molecule 1 (ICAM-1) (56). Some subsets utilize specialized mechanisms, such as melanoma cell adhesion molecule (MCAM)-expressing T helper 17 (Th17) cells, which interact with the vascular ligand lamin-411 to facilitate CNS entry (57). Furthermore, there are multiple routes of entry for lymphocytes into the CNS, including the BBB, blood–meningeal barrier (BMB), and blood–CSF barrier (BCSFB) within the choroid plexus. The latter two barriers utilize additional adhesive molecules, and entry is less stringently regulated. The BMB depends on P-selectin, which is expressed in a constitutive manner, as opposed to dynamically regulated VCAM-1 (58). The chemokine CCL20 produced by the choroid plexus can bind to the CCR6 receptor on lymphocytes, allowing entry across the BCSFB into the ventricular space (59). This provides for the preferential CNS access of CCR6^+^ subsets, such as Th17 and T regulatory (Treg) cells in the absence of the VLA-4/VCAM-1 interaction (60, 61). Since CD4^+^ anti-JCV responses in MS patients have been shown to depend primarily on Th1 cells, the reliance of this population on VLA-4/VCAM-1 contributes to the inability of natalizumab-treated patients to effectively clear JCV from the CNS (62).

Migration into the CNS is a two-step process, in which immune cells first migrate into CSF-drained perivascular, leptomeningeal, and ventricular spaces for the purpose of immunosurveillance. Interaction with antigen-presenting cells within these compartments then allows for the α6β1 integrin-mediated migration of activated lymphocytes across the glial limitans barrier into the CNS parenchyma in a cytokine-dependent manner (63). This two-step process allows for blockade of surveilling lymphocytes from the CNS parenchyma in the absence of inflammation or infection. Effective immunosurveillance, then, relies on both productive antigen presentation and activation of the appropriate lymphocyte subpopulation.

Natalizumab impairs immunosurveillance on both of these fronts through its effects on the migration and activation of T-lymphocytes and antigen-producing cells. Mature dendritic cells within perivascular spaces, meninges, and choroid plexus are important for re-stimulating peripherally activated CD4^+^ T-cells and initiating a T-lymphocyte response in the CNS (64). CD49d expression on dendritic cells varies in a maturation-dependent manner (65), such that a loss of CD49d interferes with the ability of mature dendritic cells to cross the BBB and activate T-lymphocytes (66). Furthermore, co-stimulatory signals, such as the VLA-4/VCAM-1 interaction, are necessary for effector memory T-cells to function optimally (67). Although the full extent to which natalizumab influences co-stimulatory molecule expression has not been delineated, natalizumab treatment has been shown to negatively impact surface expression of CD49d and OX40 (CD134) (68), thereby limiting T-cell activation capacity. The loss of this CNS immunosurveillance capacity is thought to contribute to the increased risk of PML for MS patients treated with natalizumab.

#### Peripheral Lymphocytes

In contrast to other MS treatments associated with PML, natalizumab leads to increases in absolute lymphocyte counts in the blood (11). This change in distribution is expected from a therapy that primarily targets the migratory capacity of lymphocytes into the CNS. However, the increased peripheral levels are not necessarily an indication that these particular populations are blocked from the CNS, since CD49d is important for adhesive interactions that mediate entry into other organs systems as well. Indeed, a direct correlation between changes in immune cells subsets between the periphery and CNS in response to natalizumab treatment has not been found. Instead, the elevated lymphocyte levels can be attributed to a selective release of lymphoid precursor cells in the hematopoietic precursor population from the bone marrow and B-lymphocytes from the spleen (69). Since natalizumab decreases surface expression of CD49d, longitudinal studies have indicated that lymphocyte subsets most dependent on CD49d for localization or trafficking are most affected, resulting in increased levels of conventional memory B-cells (51, 69, 70) and activated pro-inflammatory T-cells (68, 70–73) in the peripheral blood. This imbalance of T-cells likely occurs because certain subsets of CD4^+^ T-cells, such as Th1 cells, are particularly dependent on CD49d, whereas FoxP3^+^ regulatory T-cells express low levels of CD49d and are thus relatively unaffected by natalizumab (74). Disruption to the peripheral homeostasis of CD4^+^ T-cell populations has been proposed to contribute to the manifestation of both MS and PML, with natalizumab treatment disrupting the balance in a manner that favors the expansion of autoreactive T-cells over virus-specific T-cells (75).

A notable feature of these longitudinal studies is that the percentages of the activated subpopulations varied with duration of treatment, such that some of these changes occurred transiently at early time points, whereas others were only apparent after 1 year of treatment. This suggests that subpopulations of lymphocytes are dynamically changing in response to continued exposure to natalizumab. Since the risk of PML is typically associated with exposure to natalizumab for more than 2 years, short-term studies may not provide much insight into the relevant players conferring the increased risk, and a better understanding of the critical changes may be facilitated by long-term studies. Indeed, short-term studies have failed to detect significant changes in the peripheral CD4^+^/CD8^+^ ratio, whereas studies examining patients treated for at least 2 years found decreases in the CD4^+^/CD8^+^ ratio (76, 77). This is likely explained by the finding that while remaining within normal range, the peripheral CD4^+^/CD8^+^ ratio decreases in accordance with the number of doses of natalizumab (78). At this point, until a clear connection can be established between peripheral and CNS changes, the most meaningful predictive markers are likely to come from within the CNS.

#### CNS Lymphocytes

As expected, natalizumab treatment leads to a dramatic decline in lymphocytes within the CNS, particularly in CD19^+^ B-cells and CD4^+^ T-cells, due to their high dependency on CD49d for CNS entry (79, 80). While absolute numbers of CD8^+^ T-cells also decrease, the elevated expression of ICAM-1 and LFA-1 on these cells allows for preferential CNS access relative to CD4^+^ T-cells (81). Consequently, the CD4^+^/CD8^+^ ratio decreases toward levels seen in patients with HIV within a single dose (78), and low CD4^+^ T-cell counts have been implicated in HIV-associated PML (21). Furthermore, due to the loss of CD49d, the remaining CD4^+^ T-cells are forced to use alternative adhesion molecules, such as P-selectin glycoprotein ligand 1 and MCAM, for CNS entry (82), which will impact subset distribution and may negatively impact their functional and activation status.

In contrast to the peripheral blood, levels of pro-inflammatory chemokines and cytokines in CSF are decreased within 1 year of natalizumab treatment at the protein (83) and mRNA levels (84). This suggests that the overall milieu in the CNS is biased toward a less inflammatory state. This imbalance could be mediated by a disruption in the balance between conventional and regulatory lymphocyte subsets; however, an analysis of CSF regulatory subsets has not been performed in this context, but could be highly informative. Overall, the significant loss of CD4^+^ T-cells within the CNS coupled with the imbalances of the remaining subpopulations is likely to be a primary driver of PML risk.

#### JCV-Specific Response

The inability of natalizumab-treated PML patients to effectively clear JCV from the CNS likely stems from both the decreased migration of lymphocytes and the loss of functional capacity in the small subset of immune cells that do enter the CNS. Cellular immune response assays testing the functional capacity of JCV specific T-cells have been shown to hold predictive power for disease control in HIV-associated PML (85, 86). Consequently, some efforts have been made to determine whether measurements of the JCV-specific T-cell response also hold predictive or diagnostic power in natalizumab-associated PML. A potential caveat is the specific loss of immunosurveillance and immune function within the CNS, compared to the periphery, in natalizumab-treated patients. Unlike in HIV patients, the composition of immune cells in the CSF cannot be directly correlated to that of the peripheral blood, thus the usefulness of assays reporting the response of peripherally derived JCV specific T-cells remains unclear.

Most of this JCV-specific response testing involves a combination of intracellular cytokine staining and enzyme-linked immunosorbent assays in peripheral blood mononuclear cells (PBMCs). Consequently, the results of these studies are affected by the selection of cytokines assayed, and the *ex vivo* stimulation paradigm, particularly the JCV viral strain or peptide library, used for antigen activation. Furthermore, they are unable to assess prospective differences between patients who will eventually develop PML with those who will not and thus cannot determine the predictive value of their findings.

One pilot study found that peripheral JCV viremia was associated with decreased IFN-γ responsiveness to a JCV VP1 peptide library over the course of natalizumab treatment (87). Another study looking at JCV-specific effector memory T-cell responses found that purified JCV was a more potent antigen than VP1 alone using ELISPOT and that increased duration of treatment was associated with a stronger response (88). The probability that patients would show a positive IFN-γ response to JCV also increased the longer the patients were treated with natalizumab. Notably, stimulated PBMCs from the two PML patients in this study also exhibited robust IFN-γ responses to JCV. This suggests that while viremia and the presence of IFN-γ-producing JCV-specific effector memory T-cells in PBMCs may be indicative of peripheral JCV reactivation, they are not on their own reliable predictors of which patients will eventually develop PML. The use of more comprehensive JCV peptide libraries and cytokine assays could improve the utility of this type of testing. Indeed, another study using a more comprehensive JCV peptide library and cytokine profiling found that PML patients had aberrant responses to JCV compared to natalizumab-treated patients without PML (33). These PML patients either failed to produce a response to JCV or produced an atypical cytokine response. The CD4^+^ T-cells in two of the PML patients produced IL-10 in response to JCV, and increased levels of IL-10 were detected in the CSF from 50% of early stage PML patients. The increased CSF IL-10 may be related to the loss of CD49d^+^CD4^+^ T-cells within the CNS.

Due to differences in CD49d dependency for CNS entry, natalizumab may produce a bias toward more Treg cells and less conventional T-cells, thereby skewing the CNS cytokine milieu in a manner that facilitates viral reactivation and persistence. In a murine model of another chronic virus, y-herpesvirus, CD4^+^ T-cell deficiency led to the development of a population of IL-10-producing PD-1^+^ CD8^+^ Treg cells during viral reactivation (89). Furthermore, the loss of CD49d expression on T-cells can also impair their functional capacity. In the context of lymphatic choriomeningitis virus (LCMV) infection, virus-specific CD4^+^ T-cells have been shown to be characterized by surface expression of CDlla^hi^ and CD49d (90). Moreover, it was the CD11a^hi^ CD49d^+^ CD4^+^ memory T-cells which productively responded to a secondary challenge of LCMV, suggesting that the loss of CD49d expression on CD4^+^ T-cells could prevent an adequate immune response to JCV reactivation. Therefore, the monitoring of CD49d expression on peripheral CD4^+^ and CD8^+^ JCV-specific T-cells may provide another way to help determine PML risk.

Overall, the aberrant production of IL-10, expression of PD-1, and decreased expression of CD49d by JCV-specific T-cells may provide relevant PML risk-associated metrics although more work is needed to validate the reliability of these potential markers.

### Fingolimod

#### Mechanism

Fingolimod is an immunomodulatory drug that targets the sphingosine-1-phosphate (S1P) receptor. The loss of S1P receptors prevents lymphocytes from traversing the endothelial barriers of lymphatic compartments, effectively trapping them (91). This leads to decreases in circulating lymphocytes available to enter the CNS. The retention within lymph nodes is dependent on expression of the chemokine receptor CCR7, thus lymphocyte subsets that lack or downregulate this receptor maintain the ability to egress (92). Therefore, fingolimod disproportionally affects cell populations that express both S1P and CCR7 receptors (93). This sparing of CCR7^−^ populations was expected to mitigate the risk for PML by permitting the free migration of JCV-specific effector memory T-cells. However, PML has developed in MS patients treated with fingolimod, which may stem from recent findings that fingolimod affects effector T-cell function (94) and that CCR5^−^CCR7^+^ central memory Th1 cells play a role in the antiviral response to JCV (62). The initial cases of fingolimod-associated PML were found in MS patients who had had been previously treated with natalizumab and thus could not be conclusively linked to fingolimod (95). In recent years, there have been 15 additional MS patients where PML could be attributed to fingolimod alone, and it is important to note that there was no correlation between absolute lymphocyte count and PML onset or severity in these patients (96).

#### Peripheral Lymphocytes

In accordance with its mechanism of action, the absolute lymphocyte count in the peripheral blood decreases within 2 days of initiating fingolimod treatment, and stabilizes within 2–4 weeks, in MS patients (97). The most drastic decreases take place in the CD19^+^ B-lymphocyte and CD4^+^ T-lymphocyte populations, which have been reported to reach a steady state by 3 months of treatment (98). The distribution of the remaining B-lymphocytes is shifted toward less pro-inflammatory memory B-cells and more IL-10-producing naive B-cells and regulatory B-cells defined as CD38^+^CD24^+^ or CD38^+^CD27^-^CD24^+^CD5^+^ (99–101). This skewed cell type distribution produces an overall cytokine profile with an anti-inflammatory bias (101).

In contrast to the B-lymphocytes, the peripheral T-lymphocyte population shifts with fingolimod treatment toward a lower proportion of naive T-cells and greater proportion of memory T-cells, specifically effector memory T-cells, in both conventional and regulatory subsets (93, 98, 102), consistent with their differential expression of CCR7. Absolute levels of circulating CD4^+^ and CD8^+^ T-lymphocytes decrease in MS patients treated with fingolimod; however, CD4^+^ T-cells are affected to a greater degree, leading to a decrease in the CD4^+^/CD8^+^ ratio (93), which worsens over the course of treatment (103). The percentage of CD4^+^ and CD8^+^ T-cells that produce IFN-γ and IL-17 also decreases, primarily within the Th1 or Th1/Th17 subsets (102). Since these IFN-γ-producing populations have been shown to be critical for mounting an antiviral response to JCV (62), the fingolimod-induced decreases in these subsets has important implications for PML risk.

In addition, the relative percentage of CD25^+^CD127^−^ Treg cells within the CD4^+^ population is increased peripherally in MS patients treated with fingolimod (97, 98, 102, 104). This loss of critical IFN-γ^+^ CD4^+^ T-cells coupled with the increase in Tregs may result in the failure of the immune system to appropriately respond to pathogens in some patients.

#### CNS Lymphocytes

If the effects of fingolimod were restricted to its ability to trap particular lymphocyte populations within lymph nodes, the relative composition of lymphocytes within peripheral and CNS compartments should be similar. However, studies specifically aimed at assessing the composition of immune cells in the CNS reveal discrepancies, which suggest that fingolimod also influences migration of lymphocytes into and out of the CNS. Fingolimod-treated patients exhibit an increased CSF/blood ratio for B-lymphocytes. Interestingly, the level of IL-10-producing Breg cells (CD38^+^ CD27^−^ CD24^+^ CD5^+^) within the CSF was found not to change, despite decreases in the blood (100). The authors of this study used an *in vitro* assay to determine that B-lymphocytes, especially Breg cells, from fingolimod-treated patients have increased transendothelial migratory capacity. If B-cells are the primary carriers of JCV, this augmented ability of B-cells to cross the BBB may facilitate the entry of JCV into the CNS.

The increased migratory capacity was not found to extend to T-lymphocytes, so differences in peripheral and CNS compartments for this population may involve a different mechanism. Similar to the blood, CD4^+^ T-lymphocytes are disproportionally affected in the CSF by fingolimod treatment, but to a lesser degree (105), resulting in a decreased CD4^+^/CD8^+^ ratio, similar to what is seen in the CSF of natalizumab-treated MS patients (106). Overall, an increase in the percentage of IL-10-producing Breg cells and decrease in CD4^+^ T-cells could result in the CNS becoming an immunosuppressive environment with reduced immunosurveillance capacity, thereby allowing JCV to evade immune-mediated elimination.

#### JCV-Specific Response

Treatment with fingolimod has been associated with an increased risk for infection (107), indicating that the ability of these patients to mount an appropriate and effective immune response is compromised. Although there is currently no data regarding the effect of fingolimod on JCV-specific CTL responses, fingolimod treatment has been shown to produce a suboptimal response to another chronic latent virus, varicella zoster virus (VZV), with decreased cell proliferation and IFN-γ production (108). Notably, most fingolimod-treated MS patients exhibit a robust response to VZV, and only those who lack this response experience viral reactivation (109) The lack of response may be related to the finding that fingolimod can also impact CD4^+^ effector T-cell function through the upregulation of T-cell factor 1 (94). In this study, activated CD4^+^ T-cells were shown to produce lower levels of IFN-γ and granzyme B in the presence of fingolimod. More studies are needed to address whether a similar CTL impairment is found toward JCV in these patients.

### Dimethyl Fumarate

#### Mechanism

DMF-associated PML usually occurs in the context of severe lymphopenia, leading to the establishment of lymphocyte count guidelines for treatment discontinuation (110, 111). Five cases of PML have been reported in MS patients taking DMF (112–115). In one of these patients, lymphocyte counts did not drop below the guideline threshold for over 6 months (114). PML has also been reported in a DMF-treated psoriasis patient without severe lymphopenia (116). As a result, outside of absolute lymphocyte count, additional monitoring measures are needed.

The therapeutic mechanism of action for DMF in MS is not completely understood, but it is believed to be related to its established roles in protecting cells facing oxidative stress and blocking NF-κB-mediated pro-inflammatory cytokine production (117). The clinical benefit of fumarates in autoimmune disease is associated with their ability to disrupt the production of functional Th1 cells (117). Stimulated T-cells treated with DMF downregulate the expression of antiapoptotic Bcl-2, ultimately resulting in the loss of activated T-lymphocytes (118). The decreased differentiation of Th1 cells also appears to involve the inhibition of dendritic cell maturation, which thwarts the process of antigen presentation to T-cells (119). Furthermore, the cytokine profile of T-lymphocytes treated with DMF is altered, leading to a polarization shift away from IFN-γ and toward IL-10 (120).

#### Peripheral Lymphocytes

In a longitudinal study, the peripheral blood leukocyte and lymphocyte counts of DMF-treated MS patients decreased over a 12-month period (121). Significant loss of both central and effector memory CD4^+^ and CD8^+^ T-cells was apparent by 6 months of treatment, but CD8^+^ T-cells were more profoundly affected, leading to an increase in the CD4^+^/CD8^+^ ratio (121–124). Although among total CD4^+^ T-cells, both Th1 and Th17 classes are decreased, there is a shift away from pro-inflammatory cytokine-producing Th1 and toward anti-inflammatory cytokine-producing Tregs and Th2 cells specifically within the memory T-cell subset (123, 124). Once again, the preferential loss of Th1 cells likely impedes the production of an effective anti-JCV response, thereby increasing the risk for PML.

Similarly, DMF alters the profile of circulating B-lymphocytes in MS patients toward an anti-inflammatory state. The total levels of CD19^+^ B-lymphocytes are decreased, with the most profound loss occurring in the mature CD27^+^ B-lymphocyte subset (125–127). The proportion of Breg subsets (CD24^high^CD38^high^ and CD43^+^CD27^+^) has been found to be significantly increased after 12 months of treatment with DMF (125). Correspondingly, there is a shift in the B-cell cytokine profile toward more IL-10, relative to pro-inflammatory cytokines (125–127).

#### CNS Lymphocytes

Information on how DMF affects the composition of immune cells within the CNS is still needed. Since DMF works directly on the function and survival of the immune cells themselves, there has been an underlying assumption of a direct correlation between peripheral assays and central immune cell composition. This assumption may not be valid, based on *in vitro* studies indicating that DMF can alter cytokine-induced adhesion molecule expression in endothelial cells (128–130). DMF decreases transendothelial migration due to the downregulation of the adhesion molecules E-selectin, VCAM-1 and ICAM-1 (128). Since these adhesion molecules are important for lymphocyte trafficking into the CNS, the CNS lymphocyte composition of DMF-treated patients would be expected to differ from the periphery. In the context of experimental autoimmune encephalomyelitis (EAE), DMF also decreases the expression of CD49d on circulating lymphocytes (131), and similar to natalizumab, the loss of CD49d may prevent adequate JCV antigen presentation and clearance. If this finding applies to human MS patients, then the monitoring of CD49d expression may be prognostically useful in DMF-treated patients as well. Similar to the other PML-associated MS therapies, the ability of DMF to shift the balance between IFN-γ- and IL-10-producing cells likely inhibits the effective clearance of JCV from the CNS.

#### JCV-Specific Response

Although there is currently no information about the JCV-specific CTL response, the CD8^+^ T-cell lymphocytopenia associated with DMF suggests that the loss of JCV-specific CD8^+^ T-cells may be a contributing factor in the development of PML in lymphopenic patients. Information regarding the activation profile and responsiveness of JCV-specific CD8^+^ T-cells from DMF-treated patients will be needed to determine how this population varies over the course of treatment and whether it can be used to assay risk for PML.

## JCV Entry and Reactivation in the CNS

### Viral Entry into CNS

As a disease of the CNS, changes in lymphocyte subsets cannot lead to the development of PML if JCV is not located in the CNS. The mechanism by which JCV enters the CNS is currently unknown, but it has been proposed that it could be transferred as free virus or carried by B-cells (43). Since B-cells utilize multiple ports of entry into the CNS, there are also several avenues by which JCV could enter. JCV could be transferred directly to glial cells from B-cells that have entered the CNS parenchyma, or it could be transferred indirectly *via* the BBB, BMB, or BCSFB.

Susceptibility to infection by polyomaviruses is primarily mediated by lactoseries tetrasaccharide c (LSTc), which serves as the attachment receptor for the virus *via* the VP1 capsid protein (132, 133), and the 5-HT2 serotonin receptors, which facilitate entry (134). However, these are likely not the only mechanisms for viral entry, as some cell types prone to infection lack at least one of these receptors. Notably, glial cells, even in individuals with PML, lack expression of LSTc (135). Similarly, the LSTc expressing brain vascular endothelial cells of the BBB have been shown to be capable of supporting viral entry *in vitro*, but lack 5-HT2 receptors (136). The epithelial cells of the choroid plexus in the BCSFB express both LSTc and 5-HT2 and are thus also capable of being infected by JCV (135). Furthermore, there is clinical evidence for the productive JCV infection of the cells of the leptomeninges and choroid, resulting in meningitis (137). Interestingly, the regulatory region associated with JCV-related meningitis is not the rearranged form associated with PML, but rather the archetypal version typically found in the periphery. Thus, the NCCR rearrangements that facilitate the productive infection of oligodendrocytes are not required for JCV to enter the CNS but may play a role in the subsequent manifestation of PML.

Based on the NCCR sequence structure, four classes of JCV variants have been characterized. While particular variants are preferentially found in certain tissues, they have all been detected to some extent within both the CNS and the periphery (138). Correspondingly, latent virus has been found in the brains of healthy individuals, including both rearranged and archetypal regulatory region forms (139–141). Evidence of peripheral I-R forms, which are associated with PML and enriched in the brain, could indicate that JCV has already reached the CNS and that these patients have higher risk for PML. Therefore, prescreening of MS patients for JCV should focus not simply on seropositivity but on the detection of rearranged variants.

### JCV Reactivation Markers

Many viruses have been shown to modify the contents of host cellular secretions to promote their own spread and survival (142). Exosomes are used as a form of communication and molecular transfer between cells types and used extensively by immune cells to modulate inflammatory responses (143). The viral manipulation of exosome contents involves both altered secretion of host-derived factors and incorporation of viral-derived components (142). MicroRNAs (miRNAs) are key immunomodulatory components of exosomes. The circulating miRNA expression profile of patients with active viral infections has been shown to be altered in some cases (144). In the context of JCV infection, however, the profile of circulating host-derived miRNAs does not appear to be diagnostically useful (145). Viral-derived miRNAs, on the other hand, offer a potentially more relevant source of biomarkers, since they provide a direct readout of a specific virus. JCV encodes the miRNA jcv-miR-J1. jcv-miR-J1-5p, which is derived from the 5p arm and specific to JCV, has been detected in the urine, plasma, and CSF of JCV-seropositive individuals (146, 147). Furthermore, the presence of jcv-miR-J1-5p is indicative of latent viral infection, which may be clinically useful in the stratification of risk for PML, particularly in regards to the CSF. The miRNA derived from the 3p arm of JCV pre-miRNA, jcv-miR-J1-3p, is identical with the 3p-derived miRNA from the BK polyomavirus, so is less diagnostically useful, but important for viral immune evasion (148). JCV-miR-3p downregulates the expression of ULBP3, thereby inhibiting the clearance of virus-infected cells by natural killer cells (149). Interestingly, the results of one study suggest that due to viral homology or cross-talk, the production of antibodies toward type 1 BK polyomavirus may be protective against JCV-induced PML (150). A better understanding of JCV’s ability to modify the content and release of exosomes may allow for the development of viral reactivation diagnostic biomarkers and therapeutic interventions to prevent CNS transmission.

### Astrocytes as Gatekeepers of the CNS

Within the CNS, astrocytes are the best positioned conduit of JCV. In a chimeric mouse model, in which glia were derived from human glial progenitors, intracerebral delivery led to the preferential accumulation of JCV within astrocytes, suggesting that astrocytes are the most susceptible to JCV infection within the CNS (151). This susceptibility may stem from the vast interconnectedness of astrocytes, both with each other and with other cells in the CNS. Moreover, astrocytes play a pivotal role as the interface between the CNS and the periphery, with bidirectional communication between astrocytes and vascular endothelial cells (152). The BMB is a likely avenue of viral transmission, since meningeal cells can be productively infected by JCV *in vivo* (137), are coupled to astrocytes *via* gap junctions (153), and can influence the gap junction coupling of neighboring astrocytes (154).

Gap junction channels allow for the rapid intercellular transport of ions and small molecules, including miRNAs (155). Many pathogens have developed strategies to take advantage of gap channel-based intercellular communication to facilitate their spread (156). The release of pro-inflammatory factors results in the decreased expression of gap channel proteins, connexins, thereby disrupting networks of communication mediating essential functions, such as ionic balance, which can lead to CNS injury (157). The loss of connexins can also impair the phagocytic clearance of infected cellular debris and antigen cross-presentation at immunological synapses (156). Therefore, in addition to exosomes, viruses could potentially use gap junction channels or hemichannels to traffic viral miRNAs or proteins to neighboring uninfected cells for immunomodulation or to prime glial cells for subsequent infection.

The perivascular endfeet of astrocytes are involved in neurovascular regulation and permeability of the BBB (152). In this capacity, astrocytes serve as the gate keepers of immune cells into the CNS. The profile of chemokines released by astrocytes is tailored to the nature of the specific invading pathogen to coordinate the most effective and least damaging immune response (158). The typical response involves an initial release of pro-inflammatory molecules to recruit leukocytes into the CNS, followed by the release of more immunosuppressive factors, such as IL-10, to prevent inflammatory damage, particularly in the context of chronic infections (159, 160). Consequently, virus-mediated modification of astrocytes could impact their ability to recruit and control the T-cells necessary to clear the virus. Perhaps more importantly is the manner in which immunomodulatory therapies can disrupt the interactions between the immune system and glial cells, resulting in viral reactivation and ineffective clearance.

### JCV Infection and Reactivation within Glia

Immune cells can affect the ability of glial cells to be productively infected by JCV through cytokine-mediated changes in gene expression. JCV contains a capsid-enclosed double-stranded DNA genome that encodes early and late genes (161). The early transcript encodes the large T- and small t-antigens, which are critical for viral replication. The NCCR controls the expression of early and late viral genes through a bidirectional promoter, thus the availability of relevant transcription and mRNA-processing factors within a host cell influences the ability of the virus to propagate (162). Since T-antigen plays such a vital role in viral replication and autoregulation of the viral promoter, factors that interact or interfere with T-antigen can impact viral reactivation (163, 164).

The alternative slicing factor SRSF1 (SF2/ASF) and T-antigen are antagonistic to each other. SRSF1 inhibits the splicing of JCV pre-mRNA transcripts, which is necessary for the production of the individual viral proteins (165). Furthermore, SRSF1 also binds to the NCCR to inhibit transcription of both early and late viral genes, including T-antigen, and suppression of SRSF1 in glial cells facilitates their infection by JCV (165, 166). T-antigen, in turn, can suppress the expression of SRSF1 through interactions with the SRSF1 promoter (167). SRSF1 suppression can also be mediated by the DNA- and RNA-binding protein, Pur-alpha, through its activation of T-antigen expression and direct inhibition of SRSF1 gene expression (168). Soluble factors secreted by immune cells, such as cytokines, can tip the balance in favor of SRSF1 and suppress JCV replication. IFN-γ, specifically, has been found to inhibit T-antigen translation in a Jak/Stat-dependent manner (169). Although, the mechanism directly linking cytokines with glial SRSF1 expression is still unclear, these studies suggest that alterations in cytokine output or glial gene expression by immunomodulatory therapies could confer risk in the development of PML. Indeed, a recent 2-year longitudinal study following patients treated with natalizumab or fingolimod indicates differences in SRSF1 expression levels (170). While SRSF1 expression levels remained stable in fingolimod-treated patients, they transiently increased within a year of natalizumab and then decreased with longer duration therapy. This is consistent with the higher incidence of PML with natalizumab and increased the risk associated with long-term use. In addition, the transcription factor Spi-B, which is increased in CD19^+^ B-cells (49) and CD8^+^ T-cells (171) following natalizumab treatment, is also important for the expression of T-antigen in astrocytes (172).

The disparity of PML incidence may also be related to direct treatment-specific changes within the astrocytes themselves. While the secretion of pro-inflammatory cytokines by immune cells may be beneficial to the containment of JCV, the expression of pro-inflammatory-associated factors by astrocytes could actually contribute to JCV reactivation. A microarray analysis comparing glial cells lines resistant to JCV with those that are susceptible to JCV infection revealed that resistant cells expressed lower levels of pro-inflammatory cytokine and chemokine gene transcripts (173). This difference is related to decreased expression of the transcription factors NF-κB and NFAT4, which can modulate expression of both pro-inflammatory cytokines and JCV transcripts (174, 175). NF-κB and NFAT4 are also themselves activated by pro-inflammatory cytokines, such as TNFα, and regulated by calcium signaling/calcineurin activity in a context-dependent manner (176). These factors promote JCV early gene expression through interactions with the KB element of the NCCR, which is negatively regulated by C/EBPb (177). In addition, histone acetylation at the KB element can activate viral gene expression, and this epigenetic modification appears to involve NF-κB (178).

Therefore, decreases in pro-inflammatory signaling within astrocytes by some immunomodulatory therapies, including fingolimod and DMF, could potentially lower the risk of JCV reactivation with the CNS. Fingolimod treatment suppresses TNFα-induced pro-inflammatory genes, primarily cytokines, in human astrocytes, which confers neuroprotection (179). Critically, NF-κB nuclear translocation and signaling are also reduced (180). The stimulation of calcium-signaling/calcineurin activity by fingolimod (181) may help mediate these effects, as calcineurin has been implicated in the inhibition of NF-κB and NFAT in reactive astrocytes (176). DMF inhibits NF-κB nuclear translocation and associated pro-inflammatory cytokine expression (182–184); however, it also decreases histone deacetylase activity (185). It remains to be determined whether these or other MS therapies actually influence JCV reactivation in the astrocytes of MS patients.

Reactivation of latent JCV within astrocytes is a potential gateway to PML. Astrocytes are actively involved in oligodendrocyte survival, differentiation, and myelination through extensive cross-talk between the two glial subtypes (186). Viral components could also be transferred in the process of this communication. Due to this reliance on astrocytes, the loss or dysfunction of infected astrocytes can negatively impact oligodendrocytes and contribute to demyelinating pathology (187). Furthermore, the uptake of infected myelin and cellular debris from JCV-induced cell lysis, by phagocytic astrocytes, could facilitate the spread of the virus within the CNS (188). Overall, more studies are needed to uncover the mechanisms used by JCV to enter the CNS and the conditions governing infection and reactivation within astrocytes. The presence or absence of JCV within the CNS at the onset of immunomodulatory treatment could help further stratify patients according to which parameters are most relevant to their individual risk of developing PML. In patients with latent JCV in the CNS, the strategy should involve preventing viral reactivation. Accordingly, the type of MS therapy used should be tailored to minimize both symptoms of MS and risk of PML.

## Genetic Factors

### Viral Mutations

The development of JCV-induced CNS pathology involves genetic mutations within the viral genome. It is the nature of the mutation that determines the manner in which the infection and subsequent pathology will manifest. The mutations can affect the virus on a variety of fronts, from controlling which cell types are susceptible to infection to virulence, depending on the region of the viral genome alteration. PML is associated with complex, but highly variable, rearrangements of the NCCR, which not only facilitates infection of glial cells, but also leads to increased expression of early genes, namely T-antigen, which then boosts the replication rate (189). About half of PML patients also have mutations in the VP1 capsid protein, which can change the affinity of binding to the sialic acid containing receptors that mediate viral entry, such as LSTc, thereby increasing virulence (190). In contrast, JCV-associated diseases affecting neurons are more dependent on deletions within the late viral genes. Granule cell neuronopathy is a demyelinating disease that results from JCV infection of cerebellar granule cell neurons (191). It is associated with mutations in the *C* terminus of the JCV VP1 gene, which are believed to enhance replication in cerebellar granule cells and provide for the evasion of immune detection (192). JCV encephalitis is linked to mutations in agnoprotein (193). At this point, only three cases of JCV-induced meningitis have been reported in the literature, and the relevant mutations have not been identified (137, 194).

**Figure 2:**
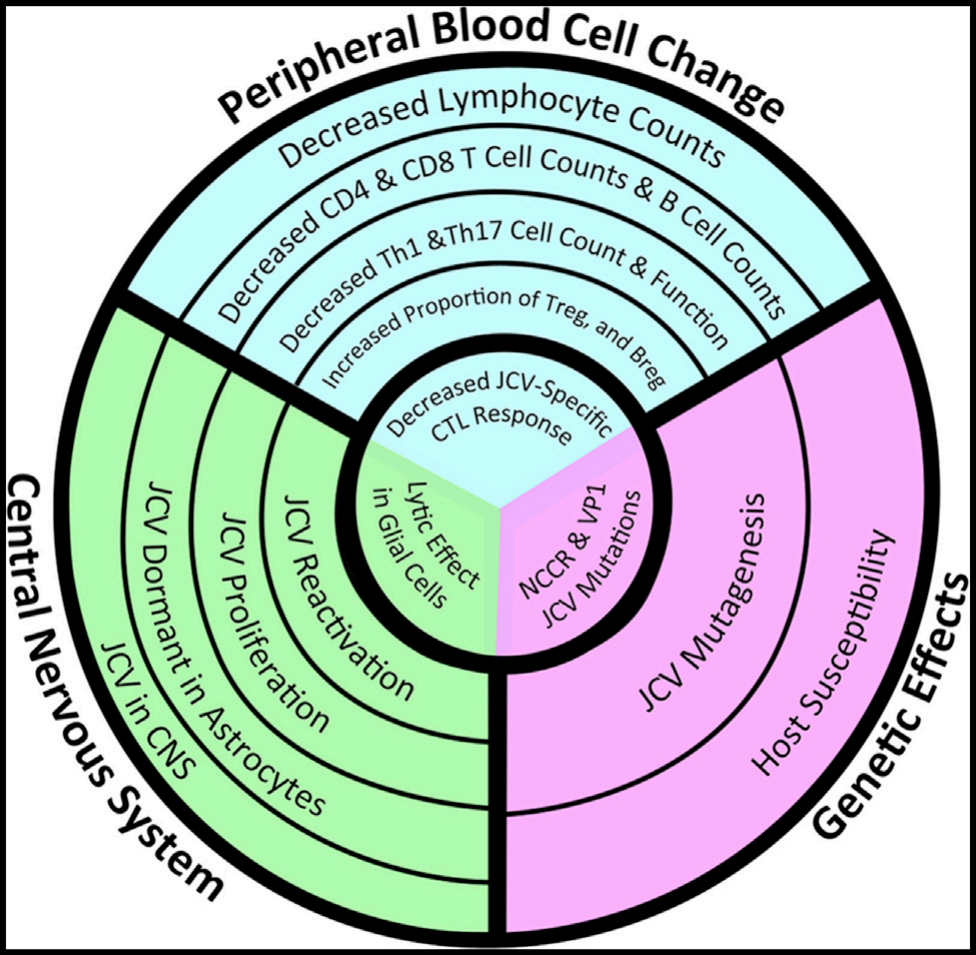
Convergence of peripheral immune changes, central nervous system (CNS) glial infectivity, and genetic susceptibility in the development of progressive multifocal leukoencephalopathy (PML). Whether a multiple sclerosis (MS) patient treated with immunomodulatory therapy will develop PML depends on changes in the peripheral immune system (blue) that lead to immunosuppression, particularly within the CNS (green), and in a genetic background (purple) that increases the susceptibility of glial cells to productive infection by John Cunningham virus (JCV). Risk increases as the circles get smaller, but a patient will not develop PML until they experience all three events located within the innermost circle: the loss of number/function of JCV-specific T-cells within the CNS in the context of a lytic JCV infection within oligodendrocytes by a version of JCV with a mutated non-coding regulatory region (NCCR). The failure of current assessments to accurately predict risk lies in the failure of these metrics to take into account all three facets. New analytics need to take into account JCV-specific lymphocyte function, JCV genetic variants, and the location of latent JCV within the body of a particular MS patient (Image copyright: Caitlyn Fisher and Yang Mao-Draayer, reprints with permission).

While PML-associated JCV may share similar features, the variability of mutations found in PML patients suggests that the viral trajectory toward a pathogenic form is unique in each individual (195). Consequently, while the loss of JCV-specific CTL and localization of JCV within the CNS are also essential features, the single greatest risk factor for PML is likely the genetic variant(s) of JCV residing within a patient (see Figure 2). This explanation best accounts for why most immunosuppressed individuals do not develop PML despite the high prevalence of (archetypal) JCV in the general population. Although individuals typically host several classes of JCV within their body, it is unclear whether the form associated with PML can be acquired through a primary infection or if it requires *de novo* mutation within the host. The rarity of PML likely reflects the complexity of the mutations needed to induce a productive infection within oligodendrocytes. The transition of the NCCR from archetypal to rearranged requires a series of deletion and duplication events, such that even if the probability of any one mutation is high, the likelihood of the combination is very low (45). However, the hijacking of host recombinase activity could bypass the requirement for several independent mutagenic events and increase the probability of pathogenic rearrangement. Currently, it is unclear if outside of immunosuppression, MS immunomodulatory therapies can directly affect the replication and mutation rates of JCV.

While immunosuppression increases viral mutation rates by facilitating viral reactivation and unchecked replication, the majority of mutations will be detrimental for the virus. Indeed, within PML patients, multiple genetic variants can be found, many of which actually make the virus less virulent (196). However, the persistence of multiple versions of the virus, even those with suboptimal adaptation, could be important for immune evasion. Recent work aimed at designing an effective vaccine for JCV suggests that the mutation of capsid proteins impairs the ability of anti-JCV antibodies to neutralize the infection (197). The presence of viral protein variants not targeted by anti-JCV antibodies could also help explain the disparity between antibody production and effective JCV clearance in PML patients. Following reactivation, continued immunosuppression further prevents the immune system from containing the mutated virus once it successfully infects oligodendrocytes. This genetic variability can also hamper analyses that rely on the detection of a specific sequence, thereby contributing to false negatives and inaccuracy of risk assessments. Efforts aimed at monitoring changes within the JCV genome in immunosuppressed patients over time could reveal the presence of a pattern of changes or cohort of mutations associated with a higher probability of developing PML. Newer assays such as high-resolution melting analysis (198) and multiplex qPCR (199) are improving the accuracy and availability of testing for NCCR rearrangements and could become a standard for monitoring risk.

### Host Genetic Susceptibility

In addition to the composition of the viral genome, the genetic makeup of the host can influence the susceptibility and response to infection. Genome-wide association studies have indicated that many single-nucleotide polymorphism (SNP) variants that confer risk toward MS and other autoimmune diseases are involved in the function of the immune system (200, 201). Consequently, some of these SNPs could also increase susceptibility or offer protection against PML. For example, SNPs that lead to the augmentation of NF-κB-mediated signaling (202) could increase the risk of viral reactivation in glial cells. In patients with these SNPs, the use of immunotherapies that specifically target NF-κB could help mitigate this risk. Human leukocyte antigen (HLA) genes encode major histocompatibility complex cell surface proteins involved in antigen presentation, which are critical for immune system regulation. The presence or absence of particular HLA alleles has been shown to confer protection or susceptibility for autoimmune disorders and infectious diseases (203). HLA-DRB1*15 is the most well-established susceptibility allele for MS (204), but may confer protection against JCV. In a study of Scandinavian and German cohorts, MS patients with the DRB1*15-DQA1*01:02-DQB1*06:02 haplotype had a negative association with JCV serostatus (205). Since elevated JCV antibody index levels are associated with increased risk for PML (15), HLA alleles associated with lower levels of JCV antibody production are predicted to minimize risk. Interestingly, there appears to be an inverse relation between HLA alleles associated with risk for autoimmune diseases and PML. HLA-DRB1*13 is negatively associated with several autoimmune diseases and is hypothesized to play a protective role (206). However, the DRB1*13-DQA1*01:03-DQB1*06:03 haplotype was positively associated with JCV serostatus (205). These findings suggest that HLA typing may be useful in the stratification of patients at risk for PML.

Variation in IL-10 signaling could also contribute to PML risk. Polymorphisms in the IL-10 promoter, which mitigate the severity of MS (207), may also increase the susceptibility for PML. In contrast, many MS patients have increased levels of miR-98 (208), which negatively regulates IL-10, and could be protective in this context. miR-98 can also inhibit IL-10 production induced by toll-like receptor 4 (TLR4) (209). In polyomavirus-susceptible mice, viral activation of B-cells by TLR4 induces an IL-10 response, whereas TLR2 activation of dendritic cells in resistant mice leads to an IL-12 response (41). This may stem from differences in the posttranscriptional stability of IL-10 mRNA downstream of the TLRs, as TLR4 has been found to protect IL-10 mRNA from degradation (210). Although TL4 is typically expressed at very low levels in human B-cells, it is increased in the context of inflammatory disease (211). Furthermore, some viruses, such as hepatitis C virus (HCV), have been shown to increase TLR4 expression on B-cells (212). In addition, TLR4 expression is downstream of IL-10, and a correlation between IL-10 promoter SNPs, TLR4 expression, and response to therapy has been found in a cohort of HCV patients (213). TLR SNPs have been hypothesized to influence infection and persistence of a variety of viruses (214), which may be related to the interaction between TLRs and IL-10. Therefore, it is possible that differences in expression of TLR4 or other TLRs on antigen-presenting cells could influence susceptibility to productive polyomavirus infection, such as JCV, in humans. However, it is unclear what effect any single SNP has on disease, which currently makes it difficult to glean reliable diagnostic metrics from host genetic data.

Case reports of supposedly immunocompetent individuals who developed PML offer some insight into which genes may be most relevant. The ability of IFN-γ to inhibit T-antigen in a Jak/Stat-dependent manner (169), suggests that its presence can help prevent JCV reactivation. Gain-of-function mutations in Stat1 increase the susceptibility of infections due to the aberrant regulation of IFN-γ-mediated inflammation (215). These mutations enhance IFN-γ-induced gene expression but impair response to IFN-γ re-stimulation. Stat1 mutations have been found in three patients who succumbed to an aggressive form of PML, and these mutations are now understood to be a major risk genetic factor for PML (216). In addition, gene sequencing from another immunocompetent PML patient revealed a genetic mutation conferring a deficit in the production of IFN-γ (217). Although even less well understood, SNPs that influence a patient’s response to immunotherapy could also impact risk. Genetic testing works best in the context of single genes with well-characterized mutations, but, for the majority of patients, susceptibility for PML will involve minor variants in a variety of genes, which may be difficult to tease out. Overall, it is likely the changes induced by a therapy in combination with the genetic makeup of the individual that determines risk.

## Conclusion

Ultimately, more studies are needed to determine why some patients develop PML. There appears to be a general scheme of immunosuppression leading to JCV reactivation and mutation toward a form that infects glia. Continued immunosuppression then inhibits effective viral clearance and culminates in PML. However, there are still many open questions regarding the details. Not all types of immunosuppression induce PML, and clearly not all immunosuppressed patients develop PML. Current studies suggest that CD4^+^ T-cell lymphocytopenia coupled with increases in IL-10-producing regulatory subsets specifically within the CNS may be most relevant. It is also unclear whether having latent JCV within the brain prior to immunosuppression confers greater risk than having it in the periphery, as well as how the mutant form of the virus develops. New biomarkers of reactivation and assays to monitor viral genomic sequences may help address these questions and provide for better monitoring strategies to reduce the future incidence of PML in MS patients.

## Author Contributions

EM and YM-D contributed to the concept, design, and writing of this review.

## Conflict of Interest Statement

EM has no conflict of interest. No commercial funding was received to support this work. YM-D has served as a consultant and/or received grant support from Acorda, Bayer Pharmaceutical, Biogen Idec, EMD Serono, Genzyme, Novartis, Questor, Chugai, and Teva Neuroscience.
